# A Clinician-Oriented Approach to Plaque Pathology in ACS: Implications for Personalized Cardiovascular Medicine—A Comprehensive Review

**DOI:** 10.3390/jpm16050240

**Published:** 2026-04-30

**Authors:** Barbara Pala, Mariagrazia Piscione, Francesco Cribari, Paola Gualtieri, Marco Alfonso Perrone, Laura Di Renzo

**Affiliations:** 1 PhD School of Applied Medical-Surgical Sciences, Tor Vergata University of Rome, Via Montpellier 1, 00133 Rome, Italy; barbara.pala93@gmail.com (B.P.);; 2 UOC Cardiologia Ospedale, Istituto Dermopatico dell’Immacolata (IDI-IRCCS), 00167 Rome, Italy; 3 Department of Cardiology, SS. Annunziata Hospital, ASL2 Abruzzo, 66100 Chieti, Italy; mariagraziapiscione@gmail.com; 4 Department of Cardiology, F. Renzetti Hospital, ASL2 Abruzzo, 66034 Lanciano, Italy; 5 Section of Clinical Nutrition and Nutrigenomics, Department of Biomedicine and Prevention, Tor Vergata University of Rome, Via Montpellier 1, 00133 Rome, Italy; 6 Division of Cardiology and CardioLab, Department of Clinical Sciences and Translational Medicine, Tor Vergata University of Rome, Via Montpellier 1, 00133 Rome, Italy

**Keywords:** plaque rupture, plaque erosion, calcific nodules, acute coronary syndromes, invasive intracoronary imaging

## Abstract

Growing evidence indicates that myocardial infarction (MI) is the clinical manifestation of heterogeneous plaque substrates with distinct molecular, cellular, and biomechanical mechanisms. Acute coronary thrombosis (ACT) most commonly arises from plaque rupture (PR), plaque erosion (PE), and calcified nodules (CNs), each associated with different inflammatory profiles, thrombus composition, clinical presentation, and prognosis. This comprehensive review provides a clinician-oriented synthesis of the pathophysiological mechanisms underlying these three principal plaque phenotypes and discusses their implications for the contemporary management of acute coronary syndromes (ACS). We examine the molecular and cellular determinants of plaque instability and highlight how systemic factors such as plaque burden, impaired healing responses, and myocardial jeopardy modulate clinical risk. The role of intracoronary and non-invasive imaging is discussed primarily as a tool to elucidate plaque biology with direct clinical relevance rather than merely as a procedural guide. Building on these insights, we propose a conceptual framework for integrating plaque biology into clinical decision-making across the acute phase, secondary prevention, and long-term follow-up. In particular, recognizing the biological heterogeneity of plaque substrates may support more personalized therapeutic strategies, enabling clinicians to tailor pharmacological and interventional approaches according to the underlying plaque phenotype and patient-specific risk profile. Finally, we briefly address emerging perspectives, including the potential role of artificial intelligence (AI) in refining plaque characterization, risk stratification, and precision cardiovascular prevention. Overall, recognition of PR, PE, and CNs as biologically distinct entities supports a shift toward mechanism-informed and personalized management of MI, aligning advances in plaque biology with the principles of precision cardiovascular medicine.

## 1. Introduction

Myocardial infarction (MI) remains one of the leading causes of morbidity and mortality worldwide, despite remarkable advances in both preventive cardiology and acute cardiovascular (CV) care [1]. In the United States alone, it is estimated that one person experiences a MI every 40 s, accounting for approximately 605,000 new and 200,000 recurrent events annually [1]. In Europe, MI incidence ranges from 43 to 144 per 100,000 person-years, with substantial variation across countries and between sexes [2]. The global burden of ischemic heart disease is expected to increase further, driven by population aging, the growing prevalence of cardiometabolic risk factors, and persistent inequalities in healthcare access [3].

The Lancet Commission proposes a paradigm shift in coronary artery disease, emphasizing that the condition should be primarily understood as a lifelong atherosclerotic process rather than a disease defined by myocardial ischaemia or acute events. Reframing coronary artery disease as atherosclerotic coronary artery disease (ACAD) supports earlier identification of disease and prioritization of preventive strategies targeting modifiable behavioural and metabolic risk factors [4]. This approach underscores the need to focus clinical efforts on the prevention, stabilization, and potential reversal of atherosclerosis to meaningfully reduce the global burden of cardiovascular morbidity and mortality.

In this context, over recent decades, robust primary prevention strategies have substantially reduced the incidence of first events, while advances in acute management—such as early reperfusion, contemporary antithrombotic regimens, and therapies targeting post-MI remodelling—have markedly improved survival and long-term outcomes [5]. Nevertheless, the relative impact, cost-effectiveness, and long-term sustainability of preventive versus therapeutic approaches remain the subject of ongoing clinical and public health debate [5].

Beyond epidemiology and treatment algorithms, growing evidence indicates that MI is not a uniform biological entity but rather the clinical expression of heterogeneous underlying plaque substrates [5]. Acute coronary thrombosis (ACT) most commonly arises from plaque rupture (PR), plaque erosion (PE), or calcified nodules (CNs), each reflecting distinct molecular, cellular, and biomechanical mechanisms [5]. These differences translate into variable inflammatory activation, thrombus composition, and degrees of coronary obstruction, ultimately influencing clinical presentation, procedural complexity, and prognosis [5]. Importantly, recognition of these biologically distinct plaque phenotypes has increasingly highlighted the need for precision and personalized approaches to cardiovascular care, in which therapeutic strategies may be tailored according to the underlying plaque biology, patient-specific risk profile, and clinical context.

Against this background, this review aims to critically examine current evidence on prevention and therapy in MI through a clinically oriented lens. We first outline the molecular and cellular pathways underpinning PR, PE, and CNs and then discuss how these pathophysiologic insights may inform clinical decision-making, including risk stratification, intensity and duration of medical therapy, and the relative role of revascularization versus optimized pharmacological management in different MI phenotypes.

Importantly, this review adopts a clinician-focused perspective, emphasizing bedside implications for secondary prevention and long-term management rather than procedural technicalities. Invasive intracoronary imaging is addressed only insofar as it clarifies biological mechanisms with direct clinical relevance, supporting a more personalized and mechanism-informed approach to the management of MI. Ultimately, integrating plaque biology, advanced imaging, and patient-level risk factors may help move contemporary cardiology toward a more individualized and precision-based management of MI.

## 2. Methods

### 2.1. Study Design

This article was conceived as a clinician-oriented narrative review aimed at integrating current knowledge on the major coronary plaque substrates underlying ACS, namely PR, PE, and CNs. A targeted literature search was performed to support a comprehensive and clinically oriented synthesis, without the intent of conducting a formal systematic or scoping review. The objective of this review was to provide a clinically oriented synthesis of mechanistic insights, imaging characteristics, and therapeutic implications associated with these distinct pathological entities.

Rather than focusing on a predefined intervention or comparative outcome, this review adopts an integrative approach that combines evidence from experimental research, translational studies, intracoronary imaging investigations, and clinical observational studies. The aim is to provide a coherent overview of the biological mechanisms and clinical relevance of different plaque phenotypes in ACS.

### 2.2. Literature Identification

The search strategy was designed to ensure representative and clinically relevant coverage of the topic, rather than to achieve exhaustive retrieval of all available studies.

Relevant literature was identified through searches of major biomedical databases, including PubMed and PubMed Central, complemented by manual screening of reference lists from key articles and authoritative reviews in the field.

The final literature search was conducted in March 2026.

**Figure 1:**
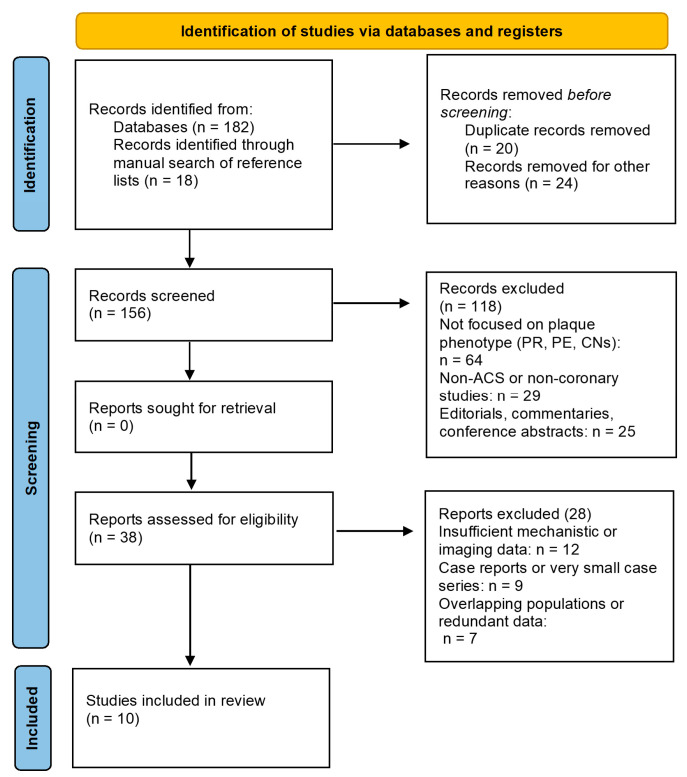
**Flow diagram of literature selection process (for transparency purposes).** Flow diagram illustrating the identification, screening, eligibility, and inclusion of studies in this systematic qualitative review according to PRISMA 2020 recommendations.

A PRISMA-style flowchart is provided solely to illustrate the literature screening process and does not indicate that a systematic review was conducted (Figure 1) [6].

A representative search strategy included combinations of the following terms:

(“plaque rupture” OR “plaque erosion” OR “calcified nodules”) AND (“acute coronary syndrome” OR “myocardial infarction”) AND (“OCT” OR “IVUS” OR “coronary imaging”).

The search strategy was designed to ensure representative and clinically relevant coverage of the topic, rather than exhaustive retrieval of all available studies.

Priority was given to studies published between January 2020 and January 2025, although earlier landmark studies and seminal pathological investigations were also considered when necessary to provide historical context and mechanistic foundations.

### 2.3. Selection of Relevant Literature

Studies were selected based on their relevance to the pathophysiology, imaging characterization, and clinical implications of coronary plaque substrates in ACS.

The review incorporates evidence from: experimental and mechanistic studies, translational cardiovascular research, intracoronary and non-invasive imaging studies, clinical observational studies and registries, systematic reviews and high-quality narrative reviews.

Case reports, editorials without original data, and studies not directly addressing coronary plaque phenotype were considered of limited relevance and were generally not included unless they provided important conceptual insights.

Study selection was therefore based on relevance and conceptual contribution, in line with the narrative and integrative nature of the review.

Study selection was performed by the authors based on clinical relevance, scientific quality, and conceptual contribution, in line with the narrative nature of the review, and was not conducted through a formal independent dual-reviewer process.

### 2.4. Data Extraction and Thematic Synthesis

Key information from selected studies was qualitatively reviewed and interpreted with particular attention to: plaque phenotype (rupture, erosion, CNs), molecular and cellular mechanisms of plaque destabilization, biomechanical and inflammatory processes involved in plaque disruption, imaging characteristics identified by intracoronary modalities (e.g., OCT, IVUS, NIRS) and non-invasive imaging techniques, clinical implications for diagnosis, risk stratification, and therapeutic strategies.

The evidence was synthesized narratively and thematically, with the discussion structured according to the major plaque substrates responsible for ACS. Particular emphasis was placed on integrating pathological findings with contemporary imaging insights and clinical management considerations.

### 2.5. Conceptual Framework

The review adopts a phenotype-oriented framework, recognizing that acute coronary syndromes arise from biologically heterogeneous plaque substrates with distinct pathophysiological pathways. By integrating pathological, imaging, and clinical evidence, this approach aims to highlight how different plaque phenotypes may influence diagnostic strategies, therapeutic decisions, and future directions in personalized cardiovascular care.

## 3. Pathophysiology of Myocardial Infarction

The pathogenesis of MI is the result of a dynamic interaction between coronary atherosclerotic plaque characteristics and systemic vulnerability [7] (Table 1). As early as the late 1980s, Müller and colleagues highlighted the role of acute external stressors—such as mental distress or physical exertion—as potential triggers of cardiac thrombotic events (CTE), noting a circadian pattern with a higher incidence in the early morning hours, coinciding with endogenous catecholamine release [7]. These observations supported the concept that ACS are often precipitated by transient triggers acting on a pre-existing vulnerable substrate, rather than arising solely from progressive luminal narrowing [7]. This framework led to the formulation of the “vulnerable plaque” concept, introduced in 1989, which describes an atherosclerotic lesion intrinsically prone to thrombosis [7]. Subsequent pathological and clinical studies have identified three principal lesion types as the dominant substrates of CTE: PR, PE and CNs [7]. Although these entities share the common final pathway of acute thrombosis, they arise from distinct biological mechanisms with important clinical implications [7].

### 3.1. Plaque Rupture

#### 3.1.1. Molecular Features of Plaque Rupture

PR represents the most extensively characterized mechanism of ACS and is typically associated with thin-cap fibroatheromas (TCFA). In these lesions, the fibrous cap—usually <65 μm in thickness—is composed primarily of extracellular matrix (ECM) proteins, particularly interstitial collagen synthesized by vascular smooth muscle cells (VSMCs) [8,9,10,11]. In TCFA, VSMCs are reduced in number, impairing collagen synthesis and limiting the capacity of the cap to maintain structural integrity [11,12].

Inflammatory activation further destabilizes the fibrous cap through macrophage-mediated degradation of ECM [11]. Activated macrophages release matrix metalloproteinases (MMPs), such as MMP-1, MMP-8, and MMP-13, which selectively degrade type I and III collagen, tipping the balance toward net collagen loss [11]. Beneath the weakened cap lies a large lipid-rich necrotic core populated by foam cells and cellular debris [8]. When cap disruption occurs, exposure of thrombogenic components such as tissue factor to circulating blood rapidly initiates thrombus formation [8,9].

#### 3.1.2. Inflammatory and Non-Inflammatory Pathways of Plaque Rupture

Autopsy studies have consistently shown that TCFA accounts for the majority of fatal MIs, underscoring the central role of structural plaque vulnerability in PR [12]. In many cases, PR is accompanied by systemic inflammatory activation, reflected by elevated C-reactive protein levels and heightened innate and adaptive immune responses [12,13]. Activated macrophages secrete proteolytic enzymes, while alterations in T-cell subsets—characterized by increased pro-inflammatory cluster of differentiation 4^+^ (CD4^+^) T cells and reduced regulatory T cells (Treg)—contribute to impaired immune regulation [14,15,16,17]. The reduction in Treg-derived cytokines such as interleukin-10 (IL-10) and transforming growth factor-β1 (TGF-β1) further amplifies plaque instability [14]. However, inflammation is not an obligatory prerequisite for PR [16]. As a matter of fact, PR may also occur in the absence of systemic inflammatory markers, driven predominantly by mechanical and neurohormonal factors [18]. Acute sympathetic activation can induce coronary vasoconstriction, increase shear stress, and generate focal mechanical strain on an already weakened fibrous cap, precipitating fissuring or rupture [19]. These observations highlight that PR is due to a wide spectrum of mechanisms ranging from inflammation-dominated to mechanically triggered events, broadening the biological landscape of acute MI [19].

#### 3.1.3. Imaging Features and Diagnostic Assessment

High-resolution intracoronary imaging has provided a crucial bridge between pathological insights and clinical practice by enabling in vivo characterization of plaque morphology in patients with ACS [20]. Modalities such as intravascular ultrasound (IVUS) and optical coherence tomography (OCT) allow identification of high-risk plaque features, including TCFA, cap disruption, and intracoronary thrombus, thereby refining risk stratification beyond angiography alone [20,21].

IVUS offers comprehensive assessment of plaque burden and vessel remodeling, parameters consistently associated with future adverse events [22]. OCT, owing to its superior spatial resolution, enables precise evaluation of fibrous cap thickness and direct visualization of rupture morphology and thrombus characteristics [23]. Rather than competing techniques, IVUS and OCT provide complementary information, integrating plaque volume, composition, and microstructural integrity [24]. Once again, in this review imaging is considered primarily as a tool to elucidate biological mechanisms with clinical relevance, rather than as a guide to procedural technique.

#### 3.1.4. Clinical Perspectives

From a clinical standpoint, PR has implications that extend beyond the treatment of the culprit lesion [25]. As demonstrated by Vergallo and colleagues in a landmark study, the presence of a TCFA at a culprit site is not an isolated phenomenon but rather a marker of a diffuse, pancoronary disease process [25]. In that study, patients presenting with a TCFA were significantly more likely to harbor additional TCFAs throughout the coronary tree, reflecting a global vulnerability of the arterial wall rather than a focal plaque abnormality [25]. This diffuse distribution of rupture-prone plaques supports the concept that MI, particularly when driven by PR, represents the clinical expression of widespread coronary instability, thereby reinforcing the need for aggressive, systemic disease-modifying therapy beyond treatment of the culprit lesion alone [26,27]. Prospective imaging studies, including PROSPECT and PROSPECT II, have demonstrated that large plaque burden, lipid-rich composition, and TCFA morphology independently predict future adverse events, even in angiographically moderate lesions. These findings support a shift from a purely stenosis-driven approach toward a biologically informed strategy that integrates plaque vulnerability into clinical decision-making [28]. While invasive imaging has primarily been studied in patients undergoing coronary angiography, advances in non-invasive coronary computed tomography angiography (CCTA) now allow assessment of high-risk plaque features—including low-attenuation plaque, positive remodelling, perivascular fat attenuation and spotty calcifications—in a broader patient population [29,30,31]. Together, intracoronary and non-invasive imaging provide complementary information that may guide the intensity of medical therapy, long-term risk stratification, and follow-up strategies in patients with high-risk coronary artery disease [31].

### 3.2. Plaque Erosion

#### 3.2.1. Prevalence and Clinical Relevance

Although PR remains the most extensively characterized mechanism of ACT, it is now well established that it does not account for all presentations of ACS [31]. A substantial proportion of patients—particularly those presenting with non ST-segment elevation myocardial infarction—experience acute coronary events in the absence of fibrous cap rupture, instead exhibiting a distinct pathological substrate known as PE [26]. Recognition of PE has challenged the traditional rupture-centric paradigm of PE since it is not merely a less severe form of PR, but a distinct entity with unique morphological, molecular, and clinical features that may influence prognosis and management [24].

#### 3.2.2. Morphological and Histopathological Features

As already said, in PR, the fibrous cap is thin and disrupted, overlying a large lipid-rich necrotic core heavily infiltrated by inflammatory cells, particularly macrophages and T lymphocytes, with extensive ECM degradation [24]. By contrast, eroded plaques are characterized by an intact and relatively thick fibrous cap, but with focal loss of the endothelial layer, leading to exposure of the subendothelial matrix to circulating blood [26]. Eroded plaques typically contain a small or absent lipid core and are enriched in vascular smooth muscle cells (VSMCs) and ECM components such as proteoglycans and hyaluronic acid (HA), with comparatively limited inflammatory infiltrate [26]. Morphologically, PE is often associated with larger residual lumen areas and less severe luminal narrowing, with more than half of lesions exhibiting <75% area stenosis [32]. Intracoronary imaging studies have further refined the phenotypic spectrum of PE, describing a continuum that includes classic fibrous erosion, thick-cap fibroatheroma (cap thickness ≥ 65 μm), and, less frequently, TCFA, underscoring the dynamic overlap between erosion- and rupture-prone lesions [32]. Thrombus composition also differs between these entities [32]. PR typically generates fibrin- and erythrocyte-rich (“red”) thrombi, whereas PE is more commonly associated with platelet-rich (“white”) thrombi [27]. OCT analyses have confirmed that white thrombus predominates at the culprit site in erosion, whereas red thrombus is more diffusely distributed along rupture-related lesions [27]. These differences have important implications for both clinical presentation and therapeutic response.

#### 3.2.3. Molecular and Pathophysiological Mechanisms

Beyond structural differences, PE is driven by distinct molecular pathways [28]. Endothelial dysfunction and detachment represent central features of PE but occur in the context of complex interactions between ECM remodelling, innate immune activation, and local hemodynamic forces [28]. A hallmark of PE is the abnormal accumulation and altered metabolism of HA at the plaque–thrombus interface [29]. HA exists in different molecular-weight forms, each conferring specific biological effects [29]. Under pathological conditions, increased activity of hyaluronidases and reactive oxygen species (ROS) promotes the generation of low-molecular-weight HA fragments, which exhibit potent pro-inflammatory and pro-thrombotic properties. These fragments stimulate VSMCs proliferation and migration, promote inflammatory cell recruitment, and induce endothelial stress and apoptosis through engagement of HA-specific receptors, including the HA-mediated motility receptor [29]. Low-molecular-weight HA also activates Toll-like receptor 2 (TLR2) signaling in endothelial cells, triggering endoplasmic reticulum stress, excessive ROS production, and release of chemokines such as interleukin-8 (IL-8) [30]. IL-8 plays a pivotal role in recruiting neutrophils and initiating the formation of neutrophil extracellular traps (NETs), complex DNA–protein structures composed of cell-free DNA, myeloperoxidase (MPO), and neutrophil elastase (NE) [30]. In the setting of PE, NETs exacerbate endothelial injury, expose pro-thrombotic substrates, and provide a scaffold for platelet adhesion and activation of the coagulation cascade [31,33]. These processes are further amplified by disturbed flow conditions, which are characteristic of arterial bifurcations where erosion preferentially occurs [31]. Oscillatory shear stress impairs endothelial integrity, promotes HA accumulation, and upregulates endothelial TLR2 expression [33]. Experimental models have demonstrated that disturbed flow alone can rapidly induce endothelial detachment, platelet adhesion, and mural thrombus formation, closely reproducing the pathological features observed in human PE [31].

Oxidized LDL accumulate within the intima and are internalized by macrophages via scavenger receptors, leading to foam cell formation, heightened local inflammation, and enhanced matrix metalloproteinase activity. The resulting lipid-rich necrotic core, attenuated fibrous cap, endothelial dysfunction, and pro-thrombotic signaling collectively increase plaque vulnerability and the propensity for erosion [34]

Moreover, adipocyte hypertrophy and immune cell dysregulation, in obese, promote a shift toward pro-inflammatory macrophage phenotypes, fostering endothelial dysfunction, lipid oxidation, and extracellular matrix remodeling. Collectively, these alterations enhance oxidative stress that may amplify the biological pathways involved in atherosclerotic plaque destabilization [35,36].

#### 3.2.4. Imaging Features and Diagnostic Assessment

Despite major advances in intracoronary imaging, in vivo diagnosis of PE remains challenging [37]. OCT cannot directly visualize the endothelial layer; therefore, the histopathological definition of erosion cannot be fully translated to imaging-based criteria [37]. IVUS and near-infrared spectroscopy (NIRS) lack sufficient resolution to reliably assess superficial plaque features. Consequently, a pragmatic imaging-based definition has been adopted in clinical practice, whereby PE is inferred when a thrombotic culprit lesion shows no evidence of fibrous cap disruption after exclusion of coronary embolism [38]. OCT-based classifications describe “definite” erosion (intact fibrous cap with overlying thrombus) and “probable” erosion (surface irregularity without visible thrombus) [38]. However, these criteria remain imperfect, as large thrombus burden may obscure an underlying rupture, and procedural factors such as thrombus aspiration can induce iatrogenic cap injury [37]. These limitations imply cautious interpretation and integration of imaging findings with clinical context.

#### 3.2.5. Clinical Perspectives

From a clinical standpoint, PE represents a distinct ACS phenotype with important therapeutic implications. Compared with PR, patients with PE are more often younger, female, and active smokers, and are less likely to exhibit hyperlipidemia or systemic inflammatory activation [30,39]. Erosion-related ACS frequently presents with preserved distal coronary flow, smaller thrombus burden, and less extensive myocardial necrosis, features that may contribute to a more favorable short-term prognosis [40]. Recognition of PE has prompted interest in alternative management strategies beyond routine stent implantation [40]. In carefully selected patients, conservative management with potent antithrombotic therapy alone has been associated with favorable clinical outcomes [40]. This strategy aims to stabilize the plaque surface (“plaque sealing”) while avoiding device-related vascular injury and long-term stent-related complications [40]. Ongoing studies are evaluating imaging-guided, individualized approaches to refine patient selection, and define the role of conservative versus invasive strategies in erosion-related ACS [41].

### 3.3. Calcified Nodules

#### 3.3.1. Morphological and Histopathological Features

CNs represent the least frequent yet clinically relevant substrate of CTE, accounting for approximately 2–7% of culprit lesions in autopsy and imaging series [42]. Histopathologically, CNs are characterized by nodular, eruptive calcific deposits that protrude into the arterial lumen through disruption of the fibrous cap [42]. They typically arise within severely calcified fibroatheromatous plaques involving both the intimal and medial layers, often containing fibrin, cholesterol clefts, and necrotic debris [42]. Two distinct morphological phenotypes of CNs have been described: eruptive and noneruptive. Eruptive CNs are defined by fragmented nodular calcium breaching the fibrous cap, frequently accompanied by endothelial denudation and overlying thrombus formation. In contrast, noneruptive CNs exhibit a thick, intact fibrous cap that separates the calcific mass from the lumen, preserving endothelial continuity and lacking associated thrombosis [43]. CNs preferentially develop at coronary hinge points—regions exposed to repetitive flexion, torsion, and elevated tensile stress—where ECM support is reduced in the proximal o middle tract of the right coronary artery [44]. In these settings, eccentric or collagen-poor calcific plates become fragile and prone to fracture [45]. Cap disruption and luminal protrusion of calcific fragments represent the hallmark of eruptive CNs and may precipitate intraplaque hemorrhage, acute luminal compromise, and clinical presentation as ACS [45,46]. Over time, healing and osteogenic remodeling of hemorrhagic and necrotic components can result in neointimal coverage and formation of noneruptive CNs, which likely represent the stabilized, mature stage of previously eruptive lesions [46].

#### 3.3.2. Molecular and Pathophysiological Mechanisms

**Figure 2:**
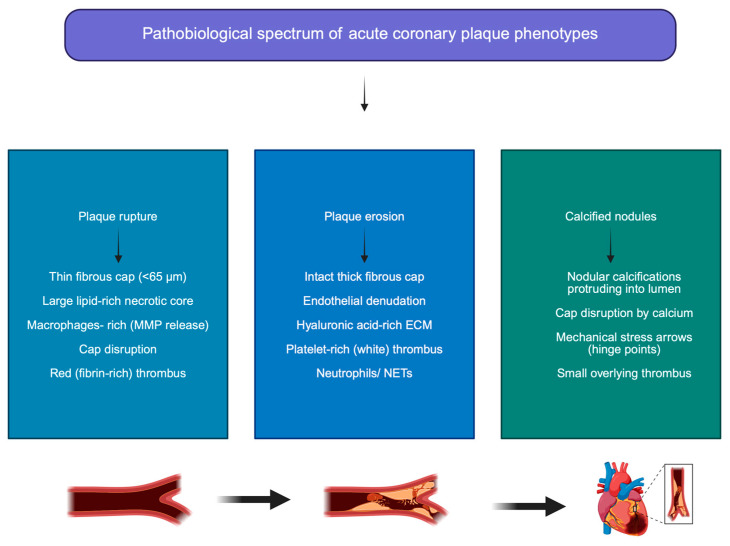
Pathobiological substrates of acute coronary thrombosis. **ECM: extracellular matrix, MMP: metalloproteinase, and NET: neutrophils extracellular traps**.

CNs reflect an advanced and largely irreversible stage of coronary atherosclerosis, arising from the interplay between mechanical stress, microfracture of calcific deposits, osteogenic differentiation, and maladaptive vascular healing [47]. Their predilection for hinge points underscores the central role of biomechanical forces in their pathogenesis [48]. In regions subjected to cyclic arterial strain, calcific deposits lacking adequate collagen scaffolding undergo repetitive microfracture, ultimately leading to disruption of the fibrous cap and extrusion of calcific material into the lumen [49]. At the molecular level, VSMCs undergo osteogenic transdifferentiation in response to oxidative stress, chronic inflammation, metabolic dysregulation, and high phosphate burden [49], a pathophysiological milieu observed in obesity and related cardiometabolic conditions, where chronic low-grade inflammation and vascular inflammatory signaling are key drivers of disease progression [13,35,36]. This phenotypic switch is driven by activation of osteogenic signaling pathways, including bone morphogenetic protein-2 (BMP-2), runt-related transcription factor 2 (Runx2), and alkaline phosphatase (ALP), promoting deposition of hydroxyapatite crystals within the vessel wall [44]. Simultaneously, endogenous inhibitors of vascular calcification become downregulated or functionally impaired, further accelerating mineral accumulation [46,47]. Recurrent intraplaque hemorrhage and microvessel rupture supply additional calcium and iron substrates, amplifying oxidative injury and facilitating mineralization of necrotic cores [48]. Progressive healing of eruptive CNs through osteogenic and fibrotic remodelling leads to neointimal encapsulation and formation of noneruptive CNs, which are mechanically rigid and biologically inert but contribute to vessel stiffening and luminal encroachment [48]. This process illustrates a continuum from mechanically triggered plaque instability to end-stage vascular sclerosis, rather than a transient phase of plaque vulnerability [48] (Figure 2).

#### 3.3.3. Imaging Features and Diagnostic Assessment

The in vivo recognition of CNs has been substantially enhanced by intracoronary imaging, which allows detailed assessment of lesion morphology and calcium distribution. On OCT, CNs appear as protruding, high-backscattering, signal-poor nodular structures with sharply defined borders and irregular luminal surfaces [48]. Eruptive CNs frequently demonstrate fibrous cap disruption, surface microfractures, and overlying thrombus, whereas noneruptive CNs present as smooth calcific prominences covered by an intact neointima [48]. IVUS, despite lower spatial resolution, remains useful for evaluating the depth and circumferential extent of calcification [49]. CNs are visualized as bright echogenic nodules with posterior acoustic shadowing that disrupt the concentric architecture of calcified plaques [50]. Near-infrared spectroscopy (NIRS) integrated with IVUS can further assist in distinguishing calcific from lipid-rich components and identifying mixed plaque phenotypes [49]. Multimodality imaging studies consistently demonstrate that CNs rarely occur in isolation, but rather coexist with diffuse, severe coronary calcification, supporting the concept that they represent focal manifestations of advanced systemic atherosclerosis [49]. We do not further elaborate on this aspect since in this review imaging is considered primarily as a means to elucidate plaque biology and clinical risk, rather than as a procedural planning tool.

#### 3.3.4. Clinical Perspectives

Clinically, CNs define a high-risk ACS phenotype associated with advanced age and a heavy burden of comorbidities, including long-standing hypertension, diabetes mellitus (DM), and chronic kidney disease (CKD) [44]. Unlike PR or PE, CNs-related ACS is predominantly driven by mechanical factors, whereby luminal protrusion of fractured calcific material initiates thrombosis rather than acute inflammatory destabilization [44]. From a management perspective, CNs are associated with both acute and long-term challenges [45]. Their rigid morphology and irregular luminal surface increase the risk of suboptimal stent expansion, malposition, restenosis, and stent thrombosis [46]. Imaging studies have consistently identified stent under-expansion as a key determinant of target lesion failure in CNs [46]. Although plaque modification techniques—such as atherectomy or intravascular lithotripsy—can improve device deliverability and acute procedural success, CNs located in tortuous or distal segments may remain difficult to treat, and incomplete calcium modification may compromise outcomes [46]. Beyond the acute phase, patients with CNs exhibit higher rates of target vessel revascularization and major adverse CV events during long-term follow-up compared with those with other plaque morphologies [46]. The natural history of CNs appears to involve repeated cycles of microfracture, thrombosis, and healing, leading to progressive luminal narrowing and arterial stiffening [46]. Collectively, these features support the view that CNs represent a mechanical endpoint of atherosclerotic remodeling, emphasizing the importance of aggressive secondary prevention and comprehensive management of systemic comorbidities rather than focal treatment alone [46].

**Table 1:** Pathobiological, imaging and clinical comparison of coronary plaque substrates in acute coronary syndromes.

Feature	Plaque Rupture (PR)	Plaque Erosion (PE)	Calcified Nodules (CNs)	References
Prevalence in ACS	Most common substrate (~60–70%)	Intermediate (~25–35%)	Rare (~2–7%)	[7,31,42]
Typical plaque morphology	Thin-cap fibroatheroma with large lipid-rich necrotic core	Intact and relatively thick fibrous cap with endothelial denudation	Nodular calcific deposits protruding into the lumen	[8,9,10,11,26,42]
Fibrous cap characteristics	Thin (<65 μm), disrupted	Thick and intact but endothelial layer lost	Disrupted by eruptive calcific fragments	[8,9,10,11,26,42]
Lipid core	Large lipid-rich necrotic core	Small or absent lipid core	Variable; often associated with heavily calcified plaques	[8,26,42]
Dominant cellular components	Macrophages, foam cells, inflammatory infiltrate	VSMCs, proteoglycans, hyaluronic acid	Osteogenic VSMCs, calcified extracellular matrix	[11,12,13,14,26,29,44]
Main molecular mechanisms	Matrix degradation by macrophage-derived MMPs; inflammatory activation	Endothelial dysfunction, HA accumulation, TLR2 signaling, NET formation	Osteogenic differentiation of VSMCs, microfracture of calcified plates, vascular mineralization	[11,12,13,14,29,30,31,32,33,47,48,49]
Thrombus composition	Fibrin- and erythrocyte-rich (“red thrombus”)	Platelet-rich (“white thrombus”)	Mixed thrombus, often associated with calcific protrusion	[27,32,42]
Typical patient profile	Older patients with high inflammatory burden and lipid-rich plaques	Younger patients, frequently smokers; more common in women	Elderly patients with severe calcification and multiple comorbidities	[30,39,44]
Common clinical presentation	STEMI or high-risk ACS	Often NSTEMI with preserved distal flow	ACS with complex calcified lesions	[30,40,44]
Imaging features (OCT/IVUS/CCTA)	Cap disruption, cavity formation, lipid-rich plaque	Intact fibrous cap with luminal thrombus, surface irregularity	Protruding calcific nodules with irregular luminal surface	[20,24,37,38,48,49,50]
Therapeutic implications	Culprit lesion PCI + aggressive systemic therapy	Selected cases may be managed conservatively with antithrombotic therapy	Often requires plaque modification (e.g., lithotripsy, atherectomy) before stenting	[26,27,28,40,46]
Prognostic implications	Marker of diffuse coronary vulnerability	Generally smaller infarcts and potentially better short-term prognosis	Higher risk of procedural complications and target lesion failure	[26,27,28,40,46]

PR: plaque rupture; PE: plaque erosion; CNs: calcified nodules; VSMCs: vascular smooth muscle cells; ECM: extracellular matrix; MMP: matrix metalloproteinase; NET: neutrophil extracellular traps; OCT: optical coherence tomography; IVUS: intravascular ultrasound; CCTA: coronary computed tomography angiography.

### 3.4. Adding Determinants of High-Risk Plaques

Beyond the specific morphological substrate, the clinical behavior of high-risk coronary plaques is strongly modulated by a series of systemic and lesion-level risk modifiers, including overall plaque burden, inflammatory activity, impaired healing responses, and the extent of myocardium at risk [7]. A large plaque burden reflects advanced, diffuse atherosclerosis and has consistently been associated with an increased likelihood of future coronary events, even in angiographically non-obstructive lesions [7]. Importantly, plaque burden serves as a surrogate of cumulative exposure to atherogenic stimuli and identifies patients in whom local plaque destabilization is more likely to translate into clinically significant events [7]. Inflammation acts as a central amplifier of plaque vulnerability by promoting endothelial dysfunction, ECM degradation, and prothrombotic activation. Systemic inflammatory activation not only accelerates fibrous cap thinning and necrotic core expansion but also lowers the threshold at which mechanical or hemodynamic stress can precipitate plaque disruption [7]. Conversely, impaired healing mechanisms—according with the double hit theory, favors the formation of layered plaques rather than adaptive remodelling [7]. Finally, the clinical impact of a high-risk plaque is critically influenced by the amount of myocardium subtended by the myocardial at risk. Plaques at the proximal site of the vessel supply a large myocardial territory and are more likely to result in extensive ischemia, hemodynamic compromise, and adverse outcomes when destabilization occurs, whereas similar lesions in smaller branches may remain clinically silent or present with limited myocardial injury [7]. The interaction between plaque biology and myocardial jeopardy therefore determines not only the likelihood of an acute event, but also its severity and prognostic implications [7]. Collectively, these modifiers underscore that plaque vulnerability is not an intrinsic, binary property of a single lesion, but the result of a dynamic interplay between local plaque characteristics and systemic host factors [7]. This perspective reinforces the need for comprehensive risk stratification and global disease-modifying strategies aimed at reducing both plaque instability and the downstream consequences of plaque-related ischemia.

## 4. From Mechanism to Management: A Clinician-Oriented Framework

Recognition of PR, PE and CNs as distinct biological substrates of MI challenges the traditional “one-size-fits-all” approach to ACS [51]. While contemporary guidelines appropriately emphasize early revascularization and standardized antithrombotic regimens, growing evidence suggests that the underlying plaque mechanism modulates ischemic risk, thrombotic behaviour, response to therapy, and long-term prognosis [51]. From a clinician’s perspective, the critical question is not whether revascularization or medical therapy is superior in absolute terms, but how these strategies should be integrated and prioritized according to plaque biology, patient phenotype, and systemic vulnerability [51].

In this context, current guideline recommendations provide an essential framework for the management of ACS; however, they are largely based on a syndrome-oriented approach and do not explicitly account for differences in plaque phenotype. A comparison between contemporary European and North American guidelines highlights both substantial alignment in core management principles and important nuances in specific therapeutic strategies [2,51]. Understanding these similarities and differences is critical to contextualize emerging plaque-informed approaches within guideline-directed care and to identify areas where future evidence may support a more individualized, mechanism-based management paradigm.

### 4.1. Comparison Between European and American Guidelines

Contemporary North American guidelines for ACS, developed by the American College of Cardiology and the American Heart Association, primarily focus on type 1 myocardial infarction, reflecting the predominance of evidence related to PR-mediated events, while type 2 myocardial infarction remains less clearly defined in terms of diagnosis, prognosis, and management [51]. These guidelines retain the traditional classification based on electrocardiographic presentation (ST-segment elevation vs. non-ST-segment elevation myocardial infarction), as emerging paradigms distinguishing occlusive from non-occlusive myocardial infarction still lack sufficient validation [51]. Compared with European recommendations, North American guidelines tend to provide less detailed justification for individual recommendations, although they incorporate a growing proportion of high-level evidence [2]. Key updates include strong support for complete revascularization in patients with multivessel disease, the confirmation of dual antiplatelet therapy as the cornerstone of ACS management with emerging evidence supporting shorter treatment strategies to reduce bleeding risk, and an increased emphasis on early and intensive lipid-lowering therapy, including the use of non-statin agents [2]. Additional updates concern transfusion thresholds and the use of mechanical circulatory support in selected patients with cardiogenic shock [2]. Overall, while largely consistent with European Society of Cardiology guidelines, North American recommendations underscore the importance of individualized treatment strategies and shared decision-making, particularly in the context of secondary prevention [2,51].

#### 4.1.1. Acute Phase: Clinical Phenotyping Beyond Stenosis Severity

In the acute setting, clinical decision-making has traditionally relied on symptoms, electrocardiographic changes, biomarker elevation, and angiographic severity of stenosis [51]. However, these parameters do not fully capture the biological heterogeneity of ACS. PR is often associated with a large necrotic core, intense inflammatory activation, and fibrin-rich thrombus, translating into extensive myocardial necrosis and higher early ischemic risk [52]. In contrast, PE typically presents with preserved vessel architecture, platelet-rich thrombus, and less extensive myocardial damage, while CNs reflect mechanically driven obstruction in the context of advanced, diffuse atherosclerosis [53]. Although definitive identification of plaque mechanism requires intracoronary imaging, clinicians can often infer the most likely substrate by integrating demographic features, risk factor profiles, inflammatory markers, angiographic appearance, and thrombotic burden. This bedside phenotyping provides a conceptual framework for tailoring therapy intensity and anticipating early and late complications, even when imaging is not routinely available [53].

#### 4.1.2. Antithrombotic Therapy: Tailoring Intensity and Duration

Antithrombotic therapy represents the cornerstone of ACS management across all plaque phenotypes; however, plaque biology may influence both thrombus composition and the balance between ischemic and bleeding risk [52,53,54,55].

PR, characterized by fibrin-rich thrombus and heightened inflammatory activation, is generally associated with a high ischemic risk, supporting the use of potent dual antiplatelet therapy and, in selected patients, consideration of prolonged antithrombotic strategies [48].

In contrast, PE is often associated with more favorable clinical and procedural characteristics. Observational and imaging-based studies have reported lower rates of major adverse cardiovascular events in PE compared with PR following PCI, both at mid- and long-term follow-up [55]. Higuma et al. showed that PE is associated with less microvascular injury and myocardial necrosis after PCI [56]. In addition, concerns have been raised regarding vascular healing after drug-eluting stent implantation in PE, with delayed endothelialization reported at short-term follow-up [57].

These findings have stimulated interest in conservative, antithrombotic-only strategies in selected patients with PE; however, current evidence remains limited and largely derived from observational and imaging-guided investigations. Prati et al. demonstrated the feasibility of deferred stenting after thrombectomy in patients with OCT-confirmed PE, reporting favorable clinical outcomes over a two-year follow-up, including in patients managed with dual antiplatelet therapy alone [58]. The EROSION study further provided prospective evidence that, in STEMI patients with OCT-defined PE, preserved distal flow (TIMI 3), and <70% residual stenosis, avoidance of stenting was associated with thrombus reduction and favorable short-term outcomes [59]. Additional OCT-guided studies have suggested that, in the absence of PR and severe luminal narrowing, a deferred or conservative strategy may allow spontaneous lumen enlargement and avoid unnecessary stent implantation without compromising short-term outcomes [60].

These observations should be interpreted with caution, as they are based on selected patient populations and specific imaging criteria, and may not be generalizable to all ACS presentations. From a clinician-oriented perspective, they support the hypothesis that a more individualized approach to antithrombotic and interventional management may be feasible in carefully selected, clinically stable patients, particularly those at increased bleeding risk; however, this concept remains hypothesis-generating and requires validation in larger randomized trials [61].

CNs represent a distinct clinical scenario [62]. In this setting, thrombosis is often driven by mechanical disruption rather than inflammatory destabilization, and patients frequently present with a high burden of comorbidities [63]. Accordingly, antithrombotic strategies should be individualized, with careful consideration of the balance between ischemic protection and bleeding risk, particularly in elderly patients and those with chronic kidney disease [64].

Overall, a plaque-informed approach may support a more nuanced application of guideline-recommended antithrombotic strategies rather than a uniform escalation of therapy; however, its integration into routine clinical practice remains dependent on further prospective validation.

#### 4.1.3. Disease-Modifying Therapy: Stabilizing the Entire Coronary Tree

**Figure 3:**
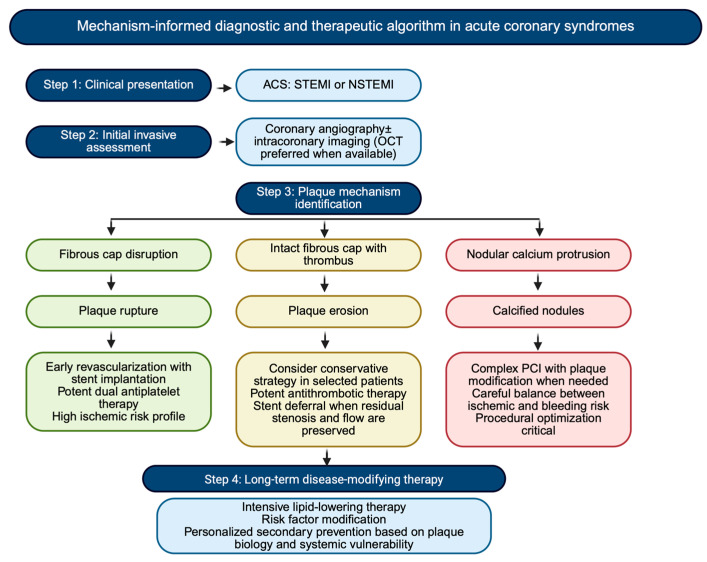
**Mechanism-informed diagnostic and therapeutic algorithm in acute coronary syndromes.** ACS: acute coronary syndrome, NSTEMI: non ST elevation myocardial infarction, OCT: optical coherence tomography, PCI: percutaneous coronary intervention, and STEMI: ST elevation myocardial infarction.

**Figure 4:**
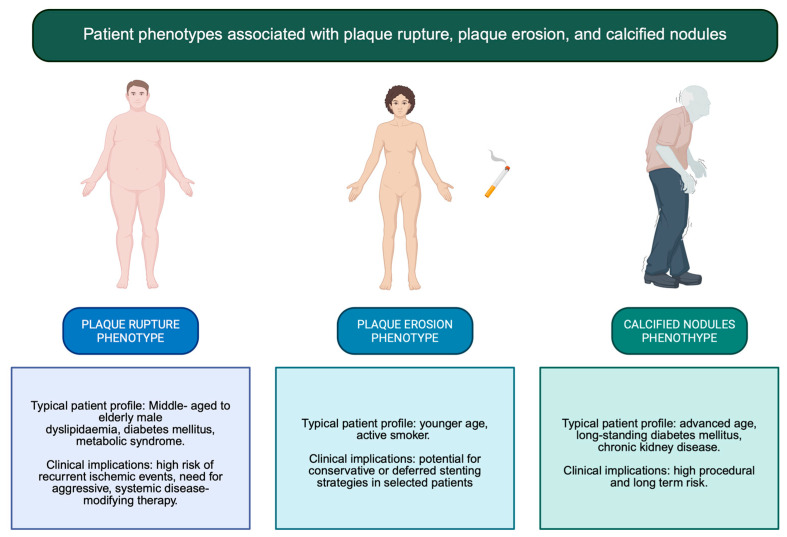
Patient phenotypes associated with plaque rupture, plaque erosion, and calcified nodules.

Regardless of the culprit mechanism, MI reflects a systemic disease process affecting the entire coronary vasculature [65]. Disease-modifying medical therapy therefore remains the most effective long-term strategy to prevent recurrence [66]. Intensive lipid-lowering therapy plays a central role in stabilizing vulnerable plaques by reducing lipid content, attenuating inflammation, and promoting fibrous cap thickening [67]. Patients with PR, in particular, may benefit from aggressive LDL-cholesterol targets and early combination therapy to mitigate residual inflammatory risk [68,69]. In PE, where dyslipidemia and systemic inflammation may be less prominent, risk factor modification focused on smoking cessation, endothelial function, and metabolic control assumes particular importance [70]. In calcified nodules, which represent an advanced stage of atherosclerotic remodeling, management should prioritize comprehensive control of cardiometabolic and mineral metabolism abnormalities, especially in patients with diabetes or CKD [71]. Across all phenotypes, anti-inflammatory strategies, blood pressure control, glycemic optimization, and lifestyle interventions complement lipid-lowering therapy and address the systemic drivers of plaque progression and destabilization [72] (Figure 3 and Figure 4).

Obesity and cardiometabolic disease are characterized by a chronic low-grade inflammatory state that promotes endothelial dysfunction, oxidative stress, and plaque destabilization. Dietary patterns strongly influence this inflammatory milieu, with pro-inflammatory diets exacerbating and anti-inflammatory dietary patterns mitigating cardiometabolic risk. Accordingly, nutritional intervention represents a fundamental disease-modifying strategy complementing pharmacological therapy, particularly in patients with obesity and metabolic dysfunction. Moreover, primary care–based clinicians occupy a pivotal role in the deployment of multimodal interventions designed to attenuate chronic inflammation, including guidance toward dietary patterns with established anti-inflammatory effects [73].

#### 4.1.4. Long-Term Follow-Up and Secondary Prevention

Long-term management after MI should reflect the underlying plaque substrate and the patient’s global risk profile [74]. Patients with PR are at heightened risk for recurrent events related to residual vulnerable plaques and may benefit from closer follow-up and sustained intensification of preventive therapy [75]. Those with PE often have a more favorable early prognosis but remain exposed to lifestyle-related triggers, underscoring the importance of behavioral interventions and adherence to antithrombotic therapy [76]. Patients with CNs represent a particularly high-risk population characterized by diffuse disease, vascular stiffness, and frequent comorbidities [76]. In these individuals, secondary prevention should extend beyond coronary events to encompass heart failure, CKD, and overall CV risk reduction.

In this context, optimized medical therapy represents a central component of long-term management and should be systematically integrated into a clinician-oriented, plaque-informed approach [2,51]. Intensive lipid-lowering therapy plays a key role in plaque stabilization, with high-intensity statins as first-line treatment and early consideration of non-statin agents, such as ezetimibe or PCSK9 inhibitors, in patients with persistent residual risk [2,51]. Antithrombotic therapy should be tailored according to the balance between ischemic and bleeding risk, while beta-blockers and renin–angiotensin system inhibitors remain essential in patients with reduced ventricular function or other guideline-based indications [2,3,4,5,6,7,8,9,10,11,12,13,14,15,16,17,18,19,20,21,22,23,24,25,26,27,28,29,30,31,32,33,34,35,36,37,38,39,40,41,42,43,44,45,46,47,48,49,50,51].

In addition, comprehensive management of cardiometabolic risk factors—including diabetes, obesity, and hypertension—together with smoking cessation and dietary interventions, is crucial to mitigate systemic inflammation and prevent recurrent plaque destabilization [2]. From a practical perspective, although plaque phenotype does not yet directly dictate specific pharmacological choices, it may help identify patients in whom greater therapeutic intensity and closer follow-up are warranted, thereby supporting a more individualized and mechanism-informed strategy for secondary prevention.

#### 4.1.5. New Perspectives and the Potential Role of Artificial Intelligence

Artificial intelligence (AI) is emerging as a promising tool to address the biological and clinical heterogeneity of MI by enabling integration of multidimensional data across scales [77]. Machine learning and deep learning approaches have demonstrated the ability to extract high-dimensional features from coronary imaging, improving plaque characterization and risk prediction beyond conventional metrics. In CCTA, AI-based models have been shown to identify high-risk plaque features and predict future events with incremental prognostic value compared with standard anatomical assessment alone [78,79,80,81,82]. Notably, recent evidence indicates that AI-enabled quantitative coronary plaque and hemodynamic analysis can further refine lesion-level risk stratification by integrating parameters such as fractional flow reserve, plaque burden, total and low-attenuation plaque volume, and myocardial blood flow, significantly improving the prediction of culprit lesions compared with conventional CCTA assessment [83].

Similarly, intracoronary imaging studies using AI-assisted OCT analysis have reported improved detection of fibrous cap thinning, PR, and erosion patterns, supporting more objective and reproducible phenotyping of culprit lesions [80,81]. In addition, recent data demonstrate that AI-based OCT analysis enables automated identification of TCFA, a key marker of plaque vulnerability, with significant prognostic implications. In a prospective cohort, AI-derived TCFA detection was associated with adverse cardiovascular outcomes and showed superior predictive performance compared with conventional core laboratory assessment, particularly when applied to the entire imaged coronary segment rather than the target lesion alone [84]. These findings highlight the potential of AI to standardize plaque characterization while improving risk stratification beyond operator-dependent analysis.

Beyond imaging, AI-driven models integrating clinical variables, biomarkers, and imaging data have been associated with enhanced prediction of recurrent ischemic events and long-term outcomes after MI, highlighting their potential role in personalized secondary prevention strategies [82].

From a clinical perspective, these advances suggest that AI-derived plaque phenotyping may contribute to more individualized decision-making, including the identification of high-risk lesions requiring intensified medical therapy and the potential selection of patients for tailored interventional versus conservative strategies. However, these applications remain largely investigational and require prospective validation. Future efforts should prioritize explainable AI frameworks, validation in diverse populations, and integration into clinician-centered workflows to ensure that AI complements, rather than replaces, clinical judgment.

## 5. Conclusions

Myocardial infarction (MI) represents the clinical expression of heterogeneous plaque substrates shaped by distinct biological and mechanical mechanisms. Recognition of PR, PE, and CNs as biologically distinct entities supports a shift toward mechanism-informed prevention and management strategies rather than a uniform treatment paradigm [70].

In this context, integrating plaque biology with systemic cardiovascular risk assessment, multimodality imaging, and optimized pharmacological therapy may allow clinicians to tailor therapeutic intensity and follow-up strategies according to the underlying disease phenotype. Advances in intracoronary and non-invasive imaging, together with emerging data-driven approaches such as artificial intelligence and quantitative plaque analysis, may further refine risk stratification and improve identification of patients at highest risk of recurrent events.

Ultimately, moving from a stenosis-centered view of coronary artery disease toward a biologically informed and patient-centered framework may enable a more personalized and sustainable strategy for secondary prevention after MI. Such an approach has the potential not only to optimize therapeutic decision-making but also to improve long-term cardiovascular outcomes through precision-guided prevention and treatment.

## Data Availability

No new data were created or analyzed in this study.

## Abbreviations

The following abbreviations are used in this manuscript:

AI	artificial intelligence
ALP	alkaline phosphatase
ACS	acute coronary syndrome
AMI	acute myocardial infarction
BMP-1	bone morphogenic protein-1
CAD	coronary artery disease
CCTA	coronary computed tomography angiography
CD4^+^	cluster of differentiation 4^+^
CKD	chronic kidney disease
CN	calcified nodules
CTE	coronary thrombotic event
CRP	C reactive protein
CTE	cardiac thrombotic events
CV	cardiovascular
DM	diabetes mellitus
ECM	extracellular matrix
HA	hyaluronic acid
HYALs	hyaluronidases
HMG-CoA	3-hydroxy-3-methylglutaryl coenzyme A
IL-8	interleukin 8
IL-10	interleukin 10
IVUS	intravascular ultrasound
LCBI	lipid core burden index
LRP	lipid-rich plaque
MI	myocardial infarction
MLA	minimal lumen area
MMPs	metalloproteinases
MPO	myelopexoxidase
NET	neutrophil extracellular traps
NIRS	near infrared spectroscopy
NE	neutrophil elastase
OCT	optical coherence tomography
PCI	percutaneous angioplasty
PE	plaque erosion
PR	plaque rupture
ROS	reactive oxygen species
Runx-2	Runt-related transcription factor-2
STEMI	ST elevation myocardial infarction
TCFA	thin-cap fibroatheromas
TGFβ1	transforming growth factor 1
Th17	T helper 17
TLR-8	Toll-like receptor 8
T_reg_	lymphocyte T regulatory cells
VH-IVUS	virtual histology intravascular ultrasound
VSMCs	vascular smooth muscle cells
